# Predictors of Swimming Ability among Children and Adolescents in the United States

**DOI:** 10.3390/sports6010017

**Published:** 2018-02-24

**Authors:** Jennifer Pharr, Carol Irwin, Todd Layne, Richard Irwin

**Affiliations:** 1 School of Community Health Sciences, University of Nevada, Las Vegas, NV 89154, USA; 2 School of Health Studies, University of Memphis, Memphis, TN 38152, USA; cirwin@memphis.edu (C.I.); telayne@memphis.edu (T.L.); 3 Office of Academic Innovation and Support Services, University of Memphis, Memphis, TN 38152, USA; rirwin@memphis.edu

**Keywords:** swimming, swimming ability, physical activity, health promotion, drowning prevention

## Abstract

Swimming is an important source of physical activity and a life skill to prevent drowning. However, little research has been conducted to understand predictors of swimming ability. The purpose of this study was to understand factors that predict swimming ability among children and adolescents in the United States (US). This was a cross-sectional survey conducted between February and April of 2017 across five geographically diverse cities. Participants were accessed through the Young Christian Men’s Association (YMCA) and included parents of children aged 4–11 years old and adolescents aged 12–17 years old. Independent *t*-test, analysis of variance (ANOVA), and univariate and multivariate analyses were conducted. Several factors were significant (*p* ≤ 0.05) predictors of swimming ability and explained 53% of the variance in swimming ability. Variables that were positively associated with swimming ability included: ability of parent(s) to swim, child/adolescent age, a best friend who enjoys swimming, water-safety knowledge, pool open all year, and encouragement to swim from parent(s). Variables that were negatively associated with swimming ability included: fear of drowning, being African American, and being female. Interventions and programs to improve the swimming ability of children and adolescents could be developed with these predictors in mind.

## 1. Introduction

Drowning is an important public health issue, especially among children and adolescents. According to the Centers for Disease Control and Prevention (CDC), there are approximately 3500 unintentional drowning deaths per year in the United States (US), with one in five drowning deaths occurring among children and adolescents aged 1–14 years [1]. For every child who dies from drowning, another five are cared for in the emergency department for a non-fatal drowning incident [1]. Additionally, racial and ethnic minority children are at greater risk for drowning than their white peers [2,3].

There are several reasons for fatal and non-fatal drowning among children and adolescents. These include a lack of: adult supervision, barriers around swimming pools, lifejackets, and swimming ability [4]. Studies have found that swimming ability is protective against fatal and non-fatal drowning in children and adolescents, and the American Academy for Pediatrics recommends that every child learns how to swim [5,6]. A study by Brenner and colleagues found that formal swimming lessons reduced the risk of drowning among children between one and four years old by 88% [6].

In addition to swimming ability being a life-saving skill, it is a good form of physical activity with many health benefits. Swimming improved peak expiratory flow in asthmatic children over other forms of sport and has been shown to improve both lung function and cardiopulmonary fitness in children and adolescents with asthma [7,8]. Swimming and other forms of aquatic exercises are ideal for children and adults with disabilities and other chronic conditions [9,10]. More generally, swimming has been shown to increase flexibility and muscular strength, decrease depression, improve mood, and improve cardiovascular fitness and agility [11,12,13,14,15,16]. Swimmers have better fitness than people who walk for exercise or people who do not exercise [11]. Additionally, people who engage in aquatic exercise have greater enjoyment and can exercise longer when compared to exercise on land [17,18].

While there have been several studies examining the physiological, biomechanical, and anthropometrical predictors of swimming performance in swimming athletes, few studies have examined predictors of swimming ability among children and adolescents [19,20,21]. Laosee and colleagues examined characteristics of children and their guardians that predict swimming ability among children in rural Thailand [22]. They found that predictors of swimming ability among children included: age of the child, formal swimming lessons, being male, guardian’s swimming ability, guardian’s income, and a self-reported life-threatening submersion experience for either the guardian or child [22]. Irwin and colleagues examined demographic characteristics that predict swimming ability among urban, minority youth [23]. They found that age, race, parent education, and income (free/reduced lunch program was used as a proxy) were associated with swimming ability.

Behavior theories such as the Social Cognitive Theory or the Ecological Model emphasize that health behavior, such as physical activity, is not just determined by individual factors but also include social and environmental factors [24,25,26]. The Social Cognitive Theory posits that health behaviors are influenced by individual factors including knowledge, self-efficacy, goals, and outcome expectations as well as environmental factors including the social environment (family and friends) and the built environment (barriers and facilitators influencing access) [24,25]. The Ecological Model suggests that physical activity is influenced by intrapersonal factors (knowledge, self-efficacy, age, etc.), interpersonal factors (family, friends, support, etc.), and community/institutional factors (e.g., access) [26]. Because of this, we examined factors within the child or adolescent, social factors including parents and friends, and factors within the built environment. The purpose of this study was to understand factors that predict swimming ability in children and adolescents in the US. We hypothesized that interpersonal factors including parents’ swimming ability, parental encouragement to swim, and income, in addition to the race/ethnicity and the age of child or adolescent, would be the strongest predictors of swimming ability.

## 2. Methods

### 2.1. Study Design and Setting

This was a cross-sectional study conducted between February and April of 2017 across five geographically diverse cities of Houston, TX, Jacksonville, FL, Las Vegas, NV, Los Angeles, CA, and Memphis, TN. These cities were selected with the assistance of the USA Swimming Foundation and the Young Men’s Christian Association (YMCA) because of the cities’ number and size of YMCA servicing different income levels (low, middle, and high) within the community, interest in participating in the research, and interest in youth swimming and drowning prevention.

The YMCA directors in each city were asked to choose at least three branches within their network, with at least one that served different income demographics (low, middle, and high income). They were also asked to administer an equitable amount of surveys across income levels to achieve a more economically balanced and characteristic sample of their metropolitan area.

### 2.2. Participants

Participants were accessed through the YMCA and included parents of children aged 4–11 years and adolescents aged 12–17 years. Parents of children 4–11 years old were asked to complete the survey for their oldest child within the age bracket. Adolescents were asked to complete the survey themselves. A research team member or trained YMCA staff member was available to help with survey completion. Participants were recruited from non-swimming programs at the YMCA (e.g., basketball, soccer, gymnastics) to reduce self-selection bias.

Parents of children were asked to read and sign a consent form prior to completing the survey. For adolescents, passive consent was achieved using a note home to parents along with follow-up electronic messages. Additionally, adolescents could decline to participate if they did not want to complete a survey.

### 2.3. Data Collection Tool

**Figure 1:**
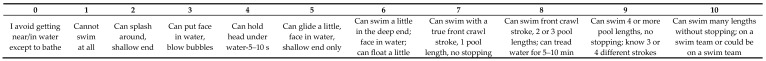
Swimming Ability Scale.

The research team had conducted similar research in the past, with a Phase I study in 2008 and a Phase II study in 2010 [23,27,28,29,30]. The survey was previously validated [27,28]. The Phase III survey (2017) was a slightly modified version of the one used in the Phase I and II studies. The survey was amended based on verbal feedback from the Phase II survey administrators and participants, results derived from statistical analyses of the previous instrument, and input from USA Swimming. The new survey was scrutinized by a panel of experts for face and content validity. The survey included four parts: (1) select demographic questions; (2) swimming ability (0–10-point scale); (3) affirmation statements (four-point Likert scale) concerning swimming; and (4) yes/no questions about facility access. The same swimming ability scale was used to assess both child/adolescent ability and parent ability (Figure 1).

The outcome variable of interest was child/adolescent swimming ability. Swimming ability was rated on a 0–10-point scale and considered to be a continuous variable. Independent variables were grouped as factors related to the child/adolescent, factors related to their parent(s)/family, or factors external to the child or parent. Independent variables are listed in Table 1. Parent swimming ability was also rated on a 0–10-point scale and considered to be a continuous variable. Child/adolescent age was between 4 and 17 years and considered to be a continuous variable. Affirmation questions that were on a four-point Likert scale (strongly disagree, disagree, agree, strongly agree) were dichotomized as disagree or agree. Race/ethnicity was categorized as Black, Hispanic, White, and other. Dummy variables were created for race/ethnicity variables and White was used as the reference. Parent education was dichotomized as less than a college degree and college degree or higher (graduate degree). Because we did not ask adolescents to report their family income, school lunch program (free/reduced price lunch or no lunch program) was used as a proxy for family income.

### 2.4. Statistical Analysis

Statistical Package for Social Sciences (SPSS) version 24 (IBM, United States) was used for the data analyses. Descriptive characteristics of the participants and mean swimming ability were generated for demographic variables. Independent *t*-tests or ANOVA were used to identify significant differences in swimming ability between demographic groups. When the ANOVA was significant, a Hochberg’s GT2 post hoc test was used to identify differences between groups because we wanted to control for type I error, there were unequal sample sizes, and we wanted a more conservative tool than Tukey-Kramer. Univariate regression analyses were conducted with each of the independent variables to determine significance. Surveys missing child/adolescent swimming ability were excluded from the regression analysis. Variables that were significant in the univariate analyses were entered into the multivariate analysis. Child/adolescent factors, parent(s)/family factors, and external factors were entered into the multiple regression in blocks. Significant variables were retained, and a final model was generated using stepwise regression to identify the best combination of predictor variables that explained the greatest amount of variability in swimming ability. Alpha was set at 0.05.

### 2.5. Ethical Approval

This study received ethical approval by the University of Memphis Institutional Review Board.

## 3. Results

In total, 1428 people participated in this study, with 600 adolescents and 828 parents included. More girls (56%) than boys (44%), African American (37%) and White (32%) participants, and participant parents having a college education (44%) were represented in the sample (Table 2).

Significant differences were observed in mean swimming ability scores, with adolescents having a higher mean score than children, and with participants whose school did not participate in free/reduced lunch programs having a higher mean score than those with free/reduced lunch programs (Table 3). ANOVA analysis and post hoc tests revealed that there were significant differences in mean swimming ability scores based on race/ethnicity, with White participants having a higher mean score than Black (*p* < 0.01), Hispanic (*p* = 0.02), or Other (*p* = 0.03) participants. Additionally, participants whose parents had an advanced college degree had a significantly higher mean swimming ability score than those whose parents had a high school diploma or General Equivalency Diploma (GED) (*p* < 0.01).

In the univariate analysis, several variables were significantly associated with swimming ability, including all child/adolescent factors, all but parent education of the parent/family factors, and all but the pool being in good condition of the external factors (Table 4). Variables that were positively associated with swimming ability included: age, enjoyment of swimming, water-safety knowledge, parent swimming ability, parent encouragement, family members knowing how to swim, no school lunch program, the nearest pool being open all year and easily accessed, and a best friend who enjoys swimming. Variables that were negatively associated with swimming ability included: being female, African American, Hispanic, or other race/ethnicity, and being afraid of getting hurt or drowning.

In the multivariate analysis using block entry, child/adolescent factors that remained significant included sex, age, African American, enjoyment of swimming, water-safety knowledge, and fear of getting hurt or drowning; these factors explained 40% of the variance in swimming ability (Table 5). Parent/family factors included parent swimming ability and encouragement to swim, which explained an additional 10% of the variance in swimming ability. External factors included the nearest pool being open all year and easily accessed, and a best friend who enjoys swimming; these factors explained an additional 3% of the variance in swimming ability.

The final model for child/adolescent swimming ability explained 53% of the variability in swimming ability (Table 6). Variables that were positively associated with swimming ability included: parental ability to swim, child/adolescent age, a best friend who enjoys swimming, water-safety knowledge, pool open all year, and encouragement to swim from parent(s). Variables that were negatively associated with swimming ability included: fear of drowning, being African American, and being female.

## 4. Discussion

Several factors that predict swimming ability were identified in this study. Parents have a strong influence on the physical activity of their children, and in this study, we found a positive relationship with children’s/adolescents’ swimming ability and their parents’ swimming ability and parental encouragement to swim. This is consistent with other physical activity research that has shown a positive association between parent-child physical activity [23,29,31,32,33]. Parents influence their children’s physical activity intensity and duration through modeling (engaging in the activity themselves) and encouragement. Additionally, parents impact adolescents’ physical activity through their attitudes towards and encouragement of physical activity [31]. Parents who swim more often have children who swim more often [29]. Because of the positive parent-child relationship with swimming, parents can be a target for interventions to increase swimming in children and adolescents by helping parents promote the importance of swimming to their children either by swimming themselves or encouraging their children to swim [31].

Children and adolescents who knew how to be safe around water and were not afraid of drowning had higher mean swimming scores. People tend to avoid activities that they either have (1) little knowledge of or (2) fear around. Many behavior theories posit that people must have knowledge about and self-efficacy (low fear) towards a behavior to engage in that behavior [24,25]. Research has shown predictors of adherence to an exercise program include exercise-specific knowledge and self-efficacy [34]. Interventions to promote swimming should teach children and adolescents water-safety knowledge and drowning prevention techniques in order to increase their self-efficacy towards swimming.

External predictors of swimming ability were friends who swim and having access to a pool year-round. Again, behavior theories such as the Social Cognitive Theory or the Ecological Model illustrate that health behaviors, including physical activity, are influenced by a person’s social and environmental factors [24,25,26]. Children and adolescents who have friends that enjoy swimming are more likely to swim with their friends. Additionally, if there is a nearby swimming pool that is open year-round, there is greater opportunity to swim. Consistent swimming improves swimming ability, and people who participate in swimming regularly are able to swim longer distances, a measure of swimming ability [16]. Directors of programs to improve swimming ability should emphasize swimming consistency and understand the importance of social relationships in the pool.

Lastly, African American children/adolescents and girls had lower swimming ability. This is consistent with previous research that found that African American girls had the highest rates of low-to-no swimming ability [23]. In addition to having lower swimming ability, African American children are at greater risk for drowning when compared to white children [2,3]. A study conducted in conjunction with the CDC found that African American children between the ages of 5 and 19 years have drowning rates that are 5.5 times greater than those of white children. Specifically, the risk of drowning is greatest for 11–12-year-old African American children, who drown in pools 10 times more frequently than white children [2]. Because swimming ability can be protective against drowning, interventions should target African American children to improve swimming ability and reduce drowning risk.

### Limitations

As with most research, this study is not without limitations and weaknesses. Subjects were recruited from YMCA, which may or may not represent the US, the city, or other YMCA populations. Additional weaknesses included self-report bias, because data were self-reported, and self-section bias, due to the nature of the participants. Although we recruited participants from non-swimming programs to reduce self-selection bias, people who chose to participate may have had a greater interest in swimming and swimming-related research. Additionally, the only way to precisely measure swimming ability is to test each person in a pool. Since this was unfeasible, we strived to accumulate a large number of responses, which has been shown to amplify validity and reliability [35,36]. The large sample size was a strength of this study.

The slightly modified research survey for Phase III was not pilot tested, as it was for Phase I and II, which was a weakness. As such, there were few changes overall, and previous survey instruments had good validity and reliability scores. Also, the pivotal swimming ability question was slightly modified for Phase III, with the addition of the first indicator, “Avoids getting near/in water except to bathe.” All subjects who chose this response were assessed on swimming ability using an additional survey question to verify they could not swim.

## 5. Conclusions

This study identified several predictors of swimming ability among children and adolescents. Predictors included factors within the child such as race/ethnicity, water-safety knowledge, and fear of drowning; factors within the parent(s), including parental swimming ability and encouragement to swim; and external factors, including a best friend that swims and year-round access to a pool. Because swimming can contribute to better health as well as being a lifesaving skill to prevent drowning, interventions to improve swimming ability of children and adolescents could be developed with these predictors in mind.

## Figures and Tables

**Table 1:** Independent Variables used for Analysis.

Child/Adolescent Factors	Parent/Family Factors	External Factors
Sex (dichotomous)	Swimming ability (continuous)	Pool is open all year (dichotomous)
Age (continuous)	Education (dichotomous)	Pool is in good condition (dichotomous)
Race (categorical/dummy variables)	School lunch program (dichotomous)	Easy access to the pool (dichotomous)
Enjoys swimming (dichotomous)	Encourages child/adolescent to swim (dichotomous)	Best friend enjoys swimming (dichotomous)
Fear of drowning (dichotomous)	Most family members know how to swim (dichotomous)	
Fear of getting hurt (dichotomous)		
Knows how to be safe around water (dichotomous)		

**Table 2:** Demographic Characteristics of Children/Adolescents.

Demographic Characteristics	4–11 Years Old (*n* = 828)	12–17 Years Old (*n* = 600)	Total (*n* = 1428)
Sex			
Male	44.6%	44.1%	44.4%
Female	55.4%	55.9%	55.6%
Race			
African American/Black	35.6%	38.3%	36.8%
Hispanic	10.1%	13.0%	11.3%
White	37.0%	25.3%	32.1%
Other	17.3%	23.3%	19.8%
Lunch Program			
Free/Reduced	39.7%	56.0%	46.0%
No Program	60.3%	44.0%	54.0%
Parent Education			
Some Elementary/Middle School	1.2%	20.1%	8.6%
Some High School	1.0%	15.3%	6.6%
High School Diploma/General Equivalency Diploma (GED)	18.0%	20.3%	18.9%
College Degree	55.6%	26.6%	44.2%
Advanced College Degree	23.8%	17.8%	21.4%

**Table 3:** Swimming Ability of Children/Adolescents by Demographic Characteristics.

Variable	*n*	Mean	SD	*p*-Value
**Age Group**				<0.01 *
4–11 years old	800	5.49	2.65	
12–17 years old	573	7.16	2.49	
**Sex**				0.56 *
Male	586	6.43	2.71	
Female	750	6.00	2.69	
**Race/Ethnicity**				<0.01 **
African American	491	5.17	2.75	
White	455	7.05	2.38	
Hispanic	159	6.34	2.53	
Other	268	6.48	2.66	
**School Lunch Program**				<0.01 *
Free/Reduced Lunch	525	5.44	2.83	
No Lunch Program	648	6.70	2.50	
**Parent Education**				<0.01 **
Some Elementary/Middle School	109	6.52	2.91	
Some High School	82	6.18	2.72	
High School Diploma/GED	244	5.62	2.98	
College Degree	577	6.01	2.64	
Advanced College Degree	278	6.77	2.39	

* = independent *t*-test, ** = ANOVA with Hochberg’s GT2 post hoc test, SD = Standard Deviation.

**Table 4:** Univariate Analysis of Factors Predicting of Swimming Ability in Children/Adolescents.

Variable	β	Standard Error	*t*	*p*-Value
**Child/adolescent factors**				
Sex (male as ref)	−0.43	0.15	−2.90	<0.01
Age	0.25	0.02	15.71	<0.01
**Race (White as ref)**				
African American	−1.88	0.17	−11.14	<0.01
Hispanic	−0.71	0.24	−2.97	<0.01
Other	−0.56	0.20	−2.82	0.01
Enjoys swimming	2.13	0.20	10.74	<0.01
Fear of drowning	−1.65	0.18	−9.06	<0.01
Fear of getting hurt	−1.25	0.19	−6.62	<0.01
Knows how to be safe around water	1.94	0.18	10.90	<0.01
**Parent/family factors**				
Parental swimming ability	0.43	0.024	17.98	<0.01
Education	−0.30	0.16	−1.89	0.06
School lunch program (no program as ref)	1.26	0.16	8.09	<0.01
Encourages child/adolescent to swim	0.45	0.17	2.68	0.01
Most family members know how to swim	1.57	0.18	8.75	<0.01
**External factors**				
Pool is open all year	0.59	0.16	3.66	<0.01
Pool is in good condition	0.55	0.31	1.79	0.07
Easy access to the pool	1.43	0.22	6.53	<0.01
Best friend enjoys swimming	1.77	0.15	11.55	<0.01

**Table 5:** Multivariate Analysis of Factors Predicting of Swimming Ability in Children/Adolescents—Block Entry.

Variable	β	Standard Error	*t*	*p*-Value
**Block 1—Child/adolescent factors (r^2^ = 0.40)**				
Sex (male as ref)	−0.35	0.13	−2.70	<0.01
Age	0.29	0.02	17.78	<0.01
**Race (White as ref)**				
African American	−0.54	0.18	−2.97	<0.01
Hispanic	0.03	0.22	0.14	0.89
Other	−0.23	0.18	−1.26	0.21
Enjoys swimming	0.50	0.22	2.26	0.02
Fear of drowning	−0.99	0.20	−4.88	<0.01
Fear of getting hurt	−0.42	0.22	−1.91	0.05
Knows how to be safe around water	0.63	0.18	3.58	<0.01
**Block 2—Parent/family factors (r^2^ = 0.50)**				
Parental swimming ability	0.29	0.03	10.47	<0.01
School lunch program	0.14	0.15	0.90	0.37
Encourages child/adolescent to swim	0.61	0.17	3.27	<0.01
Most family members know how to swim	−0.11	0.20	−0.57	0.57
**Block 3—External factors (r^2^ = 0.53)**				
Pool is open all year	0.38	0.13	2.81	<0.01
Easy access to the pool	0.46	0.20	2.33	0.02
Best friend enjoys swimming	0.69	0.16	4.42	<0.01

**Table 6:** Final Model of Factors Predicting Swimming Ability in Children/Adolescents.

Variables (r^2^ = 0.53)	β	Standard Error	*t*	*p*-Value
Parental swimming ability	0.35	0.03	10.45	<0.01
Child/adolescent age	0.35	0.03	12.76	<0.01
Best friend enjoys swimming	0.92	0.18	5.26	<0.01
Child/adolescent fear of drowning	−1.02	0.21	−4.76	<0.01
Child/adolescent knows how to be safe around water	0.58	0.20	2.89	<0.01
Pool is open all year	0.61	0.15	4.00	<0.01
African American	−0.59	0.18	−3.24	<0.01
Parent encourages child/adolescent to swim	0.76	0.28	2.73	<0.01
Child/adolescent’s sex (male as ref)	−0.34	0.15	−2.29	0.02
